# On the Antithermic, Acetanilide, as a Nerve Sedative

**Published:** 1888-03

**Authors:** 


### *ON THE ANTITHERMIC, ACETANILIDE, AS A NERVE SEDATIVE.*

Dujardin-Beaumetz, in *L'Abeille Medicale*, discusses in an interesting manner some of the therapeutic uses of acetanilide as an analgesic, and claims that it is to be placed in one group with antipyrine and phenol as analgesiacs and antipyretics.

In acetanilide, and in the new alkaloid, antipyrine, it was the antipyretic action that first attracted attention. The former is called by physicians antifebrine. Soon it was discovered that these new drugs could be employed therapeutically for other purposes than to combat pyrexia; and Dujardin-Beaumetz has demonstrated that acetanilide is particularly efficacious against the shooting pains of locomotor ataxia, those produced by compression of the nerves or cord, those which accompany neuritis of the optic nerve, *et cet.*

The dose in certain cases has been three grammes; but ordinarily one and a-half or two grammes has been administered in one day, given in one-half gramme doses. In a few cases, it produced cyanosis and chilliness, but no danger ever followed its exhibition.

Salol, which a pupil of Dujardin-Beaumetz had carefully studied, also acted as a nerve sedative, as was distinctly proven in tabes, rheumatism and other cases. It has scarcely any toxic properties at all, and can be given in doses of four to eight grammes daily.

Antipyrine is more serviceable in neuralgia, migraine and headache, while antifebrine acts better in tabes dorsalis, optic neuritis, etc.

